# DivA: detection of non-homologous and very divergent regions in protein sequence alignments

**DOI:** 10.1186/1756-0500-7-806

**Published:** 2014-11-18

**Authors:** Marie Lisandra Zepeda Mendoza, Sanne Nygaard, Rute R da Fonseca

**Affiliations:** Centre for GeoGenetics, University of Copenhagen, Copenhagen, Denmark; >Center for Social Evolution, University of Copenhagen, Copenhagen, Denmark; The Bioinformatics Centre, University of Copenhagen, Copenhagen, Denmark

**Keywords:** Multiple sequence alignment, Phylogenomic, Divergence, Homology

## Abstract

**Background:**

Sequence alignments are used to find evidence of homology but sometimes contain regions that are difficult to align which can interfere with the quality of the subsequent analyses. Although it is possible to remove problematic regions manually, this is non-practical in large genome scale studies, and the results suffer from irreproducibility arising from subjectivity. Some automated alignment trimming methods have been developed to remove problematic regions in alignments but these mostly act by removing complete columns or complete sequences from the MSA, discarding a lot of informative sites.

**Findings:**

Here we present a tool that identifies Divergent windows in protein sequence Alignments (DivA). DivA makes no assumptions on evolutionary models, and it is ideal for detecting incorrectly annotated segments within individual gene sequences. DivA works with a sliding-window approach to estimate four divergence-based parameters and their outlier values. It then classifies a window of a sequence of an alignment as very divergent (potentially non-homologous) if it presents a combination of outlier values for the four parameters it calculates. The windows classified as very divergent can optionally be masked in the alignment.

**Conclusions:**

DivA automatically identifies very divergent and incorrectly annotated genic regions in MSAs avoiding the subjective and time-consuming problem of manual annotation. The output is clear to interpret and allows the user to take more informed decisions for reducing the amount of sequence discarded but still finding the potentially erroneous and non-homologous regions.

**Electronic supplementary material:**

The online version of this article (doi:10.1186/1756-0500-7-806) contains supplementary material, which is available to authorized users.

## Findings

### Background

**Figure 1:**
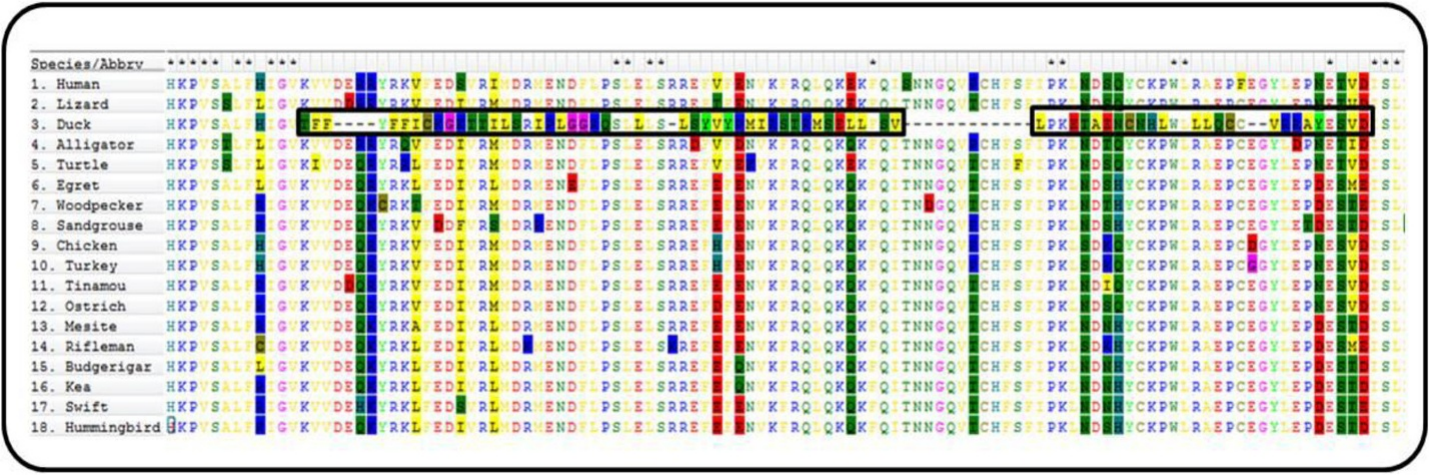
**Example of the windows identified by DivA.** Outlier windows determined by DivA are shown in black boxes. MEGA5 [10] was used to display the alignment view (the option to toggle off sites with a conservation score of more than 80% was used for an easier visualization of the outlier amino acids).

Multiple sequence alignments (MSAs) are the basis of comparative analyses that rely on sequence homology [1–4]. Alignments of homologous sequences are used to characterize protein domains, predict protein function, detect motifs and describe gene families, as well as to infer evolutionary relationships between species. However, often there are sections in MSAs that can contain sequences that are erroneously aligned. These correspond to regions that are i) under a rapid evolutionary rate, ii) non-homologous because of the choice of different splicing variants in the comparison between species, iii) wrongly annotated intron-exon barriers, iv) local structural rearrangements in a single species, etc. It is difficult to classify portions of an alignment as either very divergent or non-homologous but it has been shown that phylogenetic results are improved after removing divergent and ambiguously aligned blocks from protein sequence alignments [5]. Sequences in a MSA should be neither so similar that they are devoid of variation among the sites nor so divergent that positions are saturated by multiple substitutions, especially for phylogenetic analyses [6, 7]. Some methods have been developed to automatically clean alignments, but they mostly work by removing complete sequences if determined to be unrelated [8] or by deleting complete columns of the MSA [5]. Other approaches should be taken into account, such as the one used by Guidance [9], which can detect problematic sections of individual sequences located within regions with high alignment uncertainty (e.g. Figure 1). Alternatively, manual adjustment can be performed to remove or mask potential non-homologous regions by removing a minimum amount of sequence information, but this leads to biases and irreproducibility of the results and is impractical for large-scale genomic analyses.

**Figure 2:**
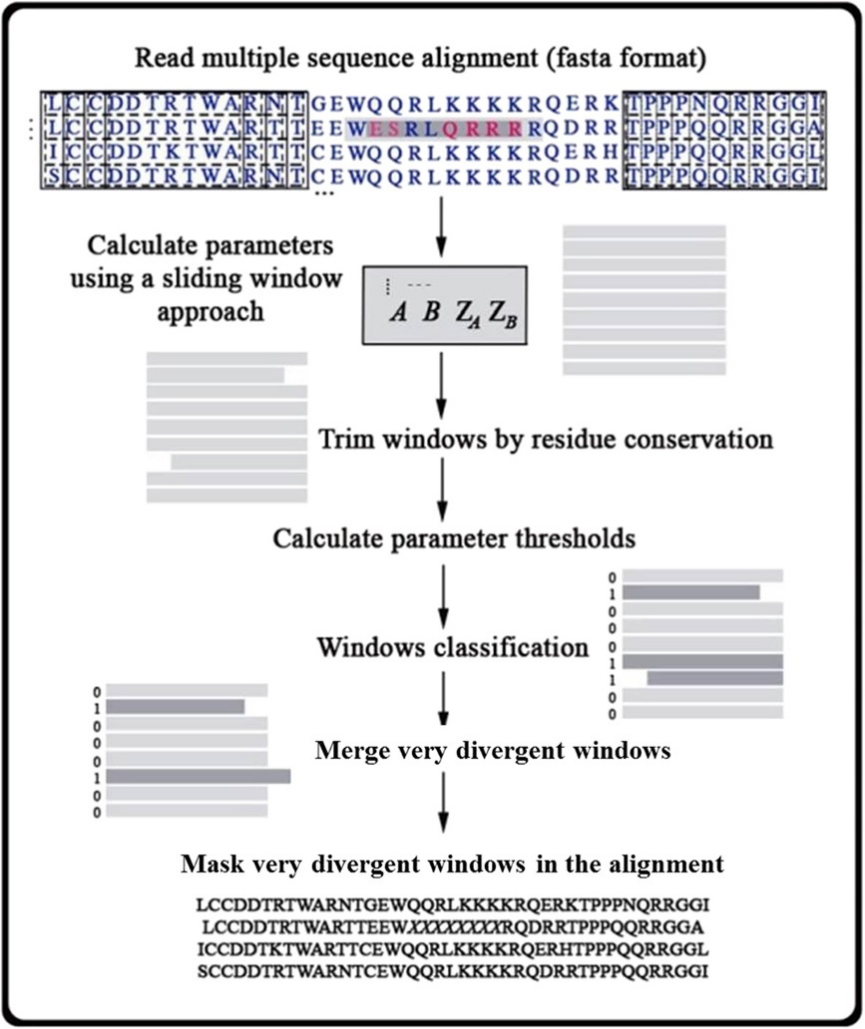
**DivA’s workflow.** Using a sliding window approach, four parameters are calculated for every sequence in every window. If there are conserved sites at the edge of each window those are trimmed and the parameter values for the window are recalculated. The threshold values for each parameter are then calculated and used to classify each window in each sequence as very divergent (potentially non-homologous) or truly homologous. Sequences from overlapping windows classified as outlier are merged, and the final coordinates are provided in the output file. The user can also obtain a new alignment file where the outlier windows are masked.

We developed DivA, a method that uses a sliding window approach to detect sections of individual sequences that are very divergent to the rest of the alignment. DivA first calculates the distribution of four parameters for each sequence in each window based on sequence similarity using both sequence weighting with position specific counts and distance-based methods. The windows for each sequence are then classified as homologous or very divergent depending on automatically calculated thresholds based on the outlier values of each parameter determined using all windows from all alignments (Figure 2). The user can change the stringency of the thresholds by choosing a desired standard deviation of the parameter values. The output is a table containing the coordinates of the very divergent windows and the values of the parameters (Table 1). Also, the user has the option to output an alignment where the outlier segments are masked.

**Table 1:** **Example of output from DivA for the Test alignment**

Alignment	Sequence	start	end	***A***	Z _***A***_	***B***	Z _***B***_
Test.fasta	Mesite	318	337	0.19	3.78	0.94	3.89
Test.fasta	Mesite	458	469	0.06	3.58	−0.80	3.86
Test.fasta	Mesite	872	882	0.22	3.68	0.20	3.92
Test.fasta	Duck	63	66	0.07	3.91	0.75	4.12
Test.fasta	Duck	564	621	0.17	4.01	0.49	4.02
Test.fasta	Duck	626	659	0.25	4.00	0.95	4.04
Test.fasta	Woodpecker	823	858	0.21	3.89	0.10	3.93
Test.fasta	Kea	768	781	0,24	3,97	0,27	4,01
Test.fasta	Ostrich	291	309	0,24	4,08	0,45	4,09

The output of the method gives information on the name of the sequence on the alignment file, the start and end positions of the very divergent window, and the four parameter values.

### Datasets

To test the performance of DivA we used 200 MSAs with sets of orthologous proteins generated by a recent avian phylogenomics project [11] and corresponding manual annotations of highly divergent sequence segments [see Additional file 1]. The 200 MSAs were chosen randomly from the 8295 orthologous sets in Jarvis *et al* [12] (this paper presents whole genome data and the corresponding annotations for 48 bird species representing 36 orders of birds). Each MSA contains protein sequences from up to 48 birds and the corresponding orthologues from the more distantly related species *Homo sapiens*, *Alligator mississippiensis*, *Chelonia mydas*, and *Anolis carolinensis* (human, alligator, turtle, and lizard, respectively). DivA was run with the default program parameters on all the alignments, both including and excluding non-birds species. We also created subsets containing 50, 100 and 200 MSAs from these 200 MSAs in which we only keep the bird sequences and exclude the sequences of the distantly related species.

### Parameter estimation

The first parameter ***A*** is based on the probability of observing the amino acids from a sequence in a given window of the alignment. The second parameter ***B*** is based on the smallest pairwise distance to another amino acid in that position calculated using blosum62 [13]. The other two parameters used are the Z-scores per sequence per window of the ***A*** and ***B*** parameters, ***Z***_***A***_ and ***Z***_***B***_, respectively. A detailed description of parameters ***A*** and ***B*** is presented next.

1. The first parameter ***A*** is based on the probability of observing the amino acids (**AA**s) in a sequence in a given window of the alignment. The probability of observing amino acid ***a*** in position ***i*** in a sequence ***S*** in an alignment corresponds to the counts for that amino acid, ***c(a***_***i***_^***S***^***)***, divided by the number of sequences ***N***: 1

The parameter ***A*** for a window of sequence ***S*** corresponds to the sum of these probabilities for each position of the window divided by the length of the window ***L***:
2

2. The second parameter ***B*** is based on the smallest pairwise distance to another amino acid in that position calculated using blosum62 [13]. The use of a column-by-column score lowers the probability of an orthologous sequence that shares high sequence similarity with different orthologs in the different sites of the window to be labeled as very divergent. For each position ***i*** of the sequence ***S***, ***a***_***i***_^***S***^ is compared to each of the amino acids on that position in the other sequences. The distance to amino acid ***b***_***i***_ in another sequence ***X*** that has the smallest dissimilarity to ***a***_***i***_^***S***^ corresponds to the highest pairwise blosum62 [13] distance: 3

The ***B*** parameter corresponds to the average of those distances:
4

For the calculation of the parameters, sequence segments were discarded when presenting more than 40% gaps or a ***P(a***_***i***_^***S***^***)*** <70%, indicative of weakly conserved sequences. In order to ensure that only the smallest required amount of sequence is discarded, if the probability of observing the first or last amino acid in a given window of a given sequence is higher than 0.9 that amino acid is removed and the parameters are re-calculated for the resized window.

### Determination of parameter thresholds

**Figure 3:**
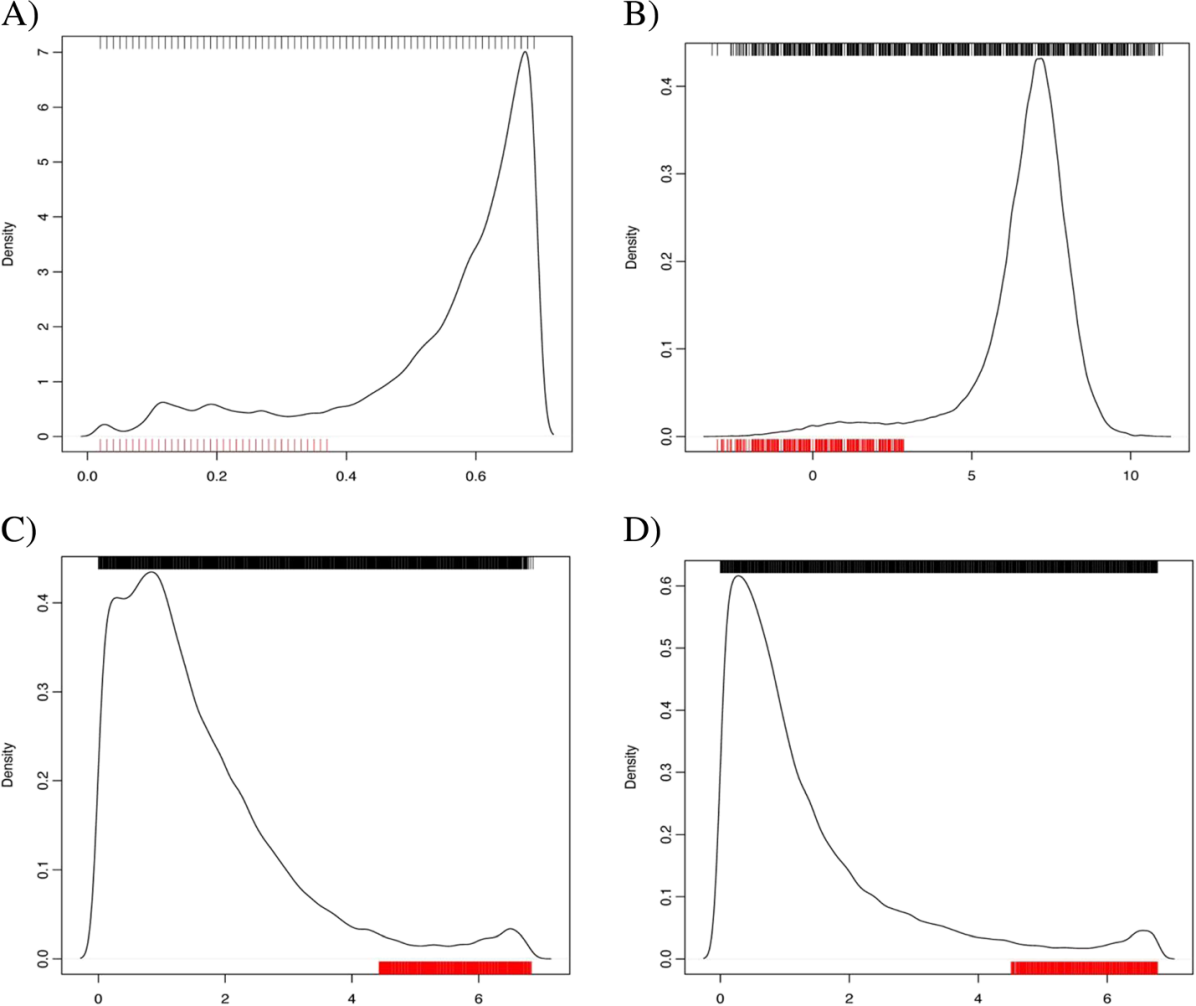
**Distributions of the parameter values calculated for the 200 bird-only alignments.** The upper ticks show the values of the homologous windows and the ticks on the lower X-axis show the values of the outlier windows. **A)** Distribution of the ***A*** parameter. **B)** Distribution of the ***B*** parameter. **C)** Distribution of the ***Z***_***A***_ parameter. **D)** Distribution of the ***Z***_***B***_ parameter.

We started by analyzing the distribution of the four parameters values calculated for the 200 bird-only alignments (Figure 3). The distribution of the values on the very divergent and homologous windows is not clearly differentiated, with some values of some parameters from very divergent windows overlapping values from homologous windows, thus posing difficulties for a straightforward thresholds definition. For the calculation of the parameter thresholds, sequence segments were discarded when presenting more than 40% gaps, indicative of very conserved sequences. The outliers were defined in terms of the Z scores for all four parameters: *Z(****A****)* < 1, *Z(****B****)* < 2, *Z(****Z***_***A***_*)* > 2, and *Z(****Z***_***B***_*)* > 2. We used the decision tree method from the R package ‘tree’ [14] to find the thresholds that define an outlier sequence segment according to the manual annotation (Additional file 2: Figure S1A).

We performed a 10-fold cross-validation with half of the dataset using the prune.tree function and the misclass method in R (package ‘tree’). This showed a low misclassification error rate (1.3e-05) and residual mean deviance (3.2e-04) (Additional file 2: Figure S1B and S2C). Furthermore, we used the other half of the dataset as validation set with the predict function, and classified it with the previously defined thresholds. In agreement with the previous result, we obtained a low misclassification rate of 1.3e-05 (Additional file 2: Figure S1D and S2E). The misclassification rate was calculated as the number of misclassifications divided by the total number of data points in the validation dataset.

### Guidance performance comparison to DivA

Guidance [9] is a currently available program for detecting problems in single sequences in a MSA; it works by assigning a confidence value for every position of the alignment. We compared the output from Guidance and that from DivA using the dataset of 200-only bird alignment and the dataset of 200 alignments including the very divergent species. In Guidance two thresholds of confidence values were used; one considering the window as very divergent and potentially non-homologous if having a Guidance score equal or less than 0.4; and a second very relaxed one that considers the window as such if the threshold is less than 0.8.

## Results and discussion

To test the impact of the size of the dataset, DivA was applied to the datasets of 50, 100, and 200 only-birds alignments. Efficiency tests were applied to the classified windows and the results show that the model performs best with big datasets (Table 2), as expected in a phylogenomics analysis where up to thousands of alignments are concatenated (Additional file 2: Figure S2). We further explored the impact of the divergence to the classification and showed that DivA has a very high sensitivity (81% TPR) for the bird-only alignments, compared to the alignments including other vertebrate species (47% TPR) (Table 2). This can be explained by the increasing difficulty in distinguishing between true divergence and error. We also examined the divergence in the alignment with the highest number of TP very divergent windows and the one with highest number of FP very divergent windows from the 200 only-birds alignments (Additional file 2: Table S1).

**Table 2:** **Dataset impact on model accuracy**

MSAs dataset	TPR	FDR	PPV
50 only-bird	0.7970402	0.6267327	0.3732673
100 only-bird	0. 812071	0. 4976526	0. 5023474
200 only-bird	0. 810281	0. 3775697	0. 6224303
200 all species	0. 469429	0. 5588211	0. 4411789

The table shows the efficiency tests results on different datasets with different sizes (50 MSAs, 100, and 200) and divergence (only birds, and birds plus distant species). True positives (TP) correspond to the number of alignment positions included in outlier windows by DivA that were also detected to be outlier by the manual annotation. False positives (FP) are located within outlier windows but were not contemplated in the manual annotation. False negatives (FN) were manually annotated as outlier, but were not detected by DivA as such. True negatives (TN) are absent in windows annotated as outlier both manually and using DivA. TPR: true positive rate, FDR: false discovery rate, PPV: positive predictive value.

To our knowledge, the only other method currently available for detecting problems in single sequences in a MSA is Guidance [9], but it does so only when the sequence segment is located in a region of high alignment uncertainty (Additional file 2: Figure S3). The comparison of the performance of Guidance and DivA showed that DivA produced better efficiency test results for the two datasets used with the two score thresholds in Guidance (Table 3).

**Table 3:** **Efficiency tests of Guidance and DivA**

	TPR		FDR		PPV	
Method	200 only birds	200 all species	200 only birds	200 all species	200 only birds	200 all species
Guidance Score <=0.4	0.3687836	0.3687836	0.9084643	0.9084643	0.09153575	0.09153575
Guidance Score <=0.8	0.5439066	0.7788185	0.928089	0.8882818	0.07191096	0.1117182
DivA	0. 810281	0. 469429	0. 3775697	0. 5588211	0. 6224303	0. 4411789

Guidance performance on the datasets of 200 only-birds alignments and the 200 alignments with very divergent species included was compared to DivA. Two threshold values, 0.4 and 0.8, were used in Guidance to consider a sequence region as potentially non-homologous.

## Conclusions

The present method was developed to solve the subjective and time-consuming problem of manual annotation and identification of incorrect gene annotation in genomic projects with phylogenomic studies. It uses a statistical framework that takes into account the next information: i) probability of an amino acids appearing in a position in the window alignment, ii) the smallest pairwise distance to another amino acid in that position in the window, and iii) the Z-score of i) and ii). That information is then integrated into a binary decision making model for the window to be classified as very divergent or truly homologous. It is easy to use; it does not require a manual annotation or input training set, and its parameter values are obtained automatically. The output is clear to interpret and allows the user to take more informed decisions for reducing the amount of sequence discarded but still finding the potentially erroneous and non-homologous regions.

## Availability and requirements

- ● Project name: DivA
- ● Project home page: https://github.com/lisandracady/DivA
- ● Operating system(s): Platform independent
- ● Programming language: Python 2.7
- ● Other requirements: Python packages numpy, re, os, sys, argparse, Bio
- ● License: Lesser GPL 3 (LGPL 3)
- ● Any restrictions to use by non-academics: None.

### Availability of supporting data

The data sets supporting the results of this article are available in the “The avian phylogenomic project” data repository, http://gigadb.org/dataset/101000. In particular, the ones used in this article are in https://github.com/lisandracady/DivA/tree/master/MUSCLEalns and https://github.com/lisandracady/DivA/tree/master/MUSCLE_birdsOnly.

## Electronic supplementary material

Additional file 1: **Manual annotation.** This file contains the manually annotated very divergent regions of the 200 alignments including the very divergent species. (CSV 13 KB)

Additional file 2: **Additional figures and table.** This file contains the supplementary figures and the supplementary table referenced in the main text. (PDF 530 KB)

## Abbreviations

MSA: Multiple sequence alignment
TP: True positive
FP: False positive
FN: False negative
TN: True negative
TPR: True positive rate
TNR: True negative rate
PPV: Positive predictive value
SD: Standard deviation.
