# Supplementary material for: DivA: detection of non-homologous and very divergent regions in protein sequence alignments
Source: BMC Res Notes. 2014 Nov 18;7:806. doi: 10.1186/1756-0500-7-806 (PMC4240845; doi:10.1186/1756-0500-7-806)
Supplement: Supplementary file 2 — Additional file 2: Additional figures and table. This file contains the supplementary figures and the supplementary table referenced in the main text. (PDF 530 KB) [file 13104_2014_3322_MOESM2_ESM.pdf]

Additional figures and table

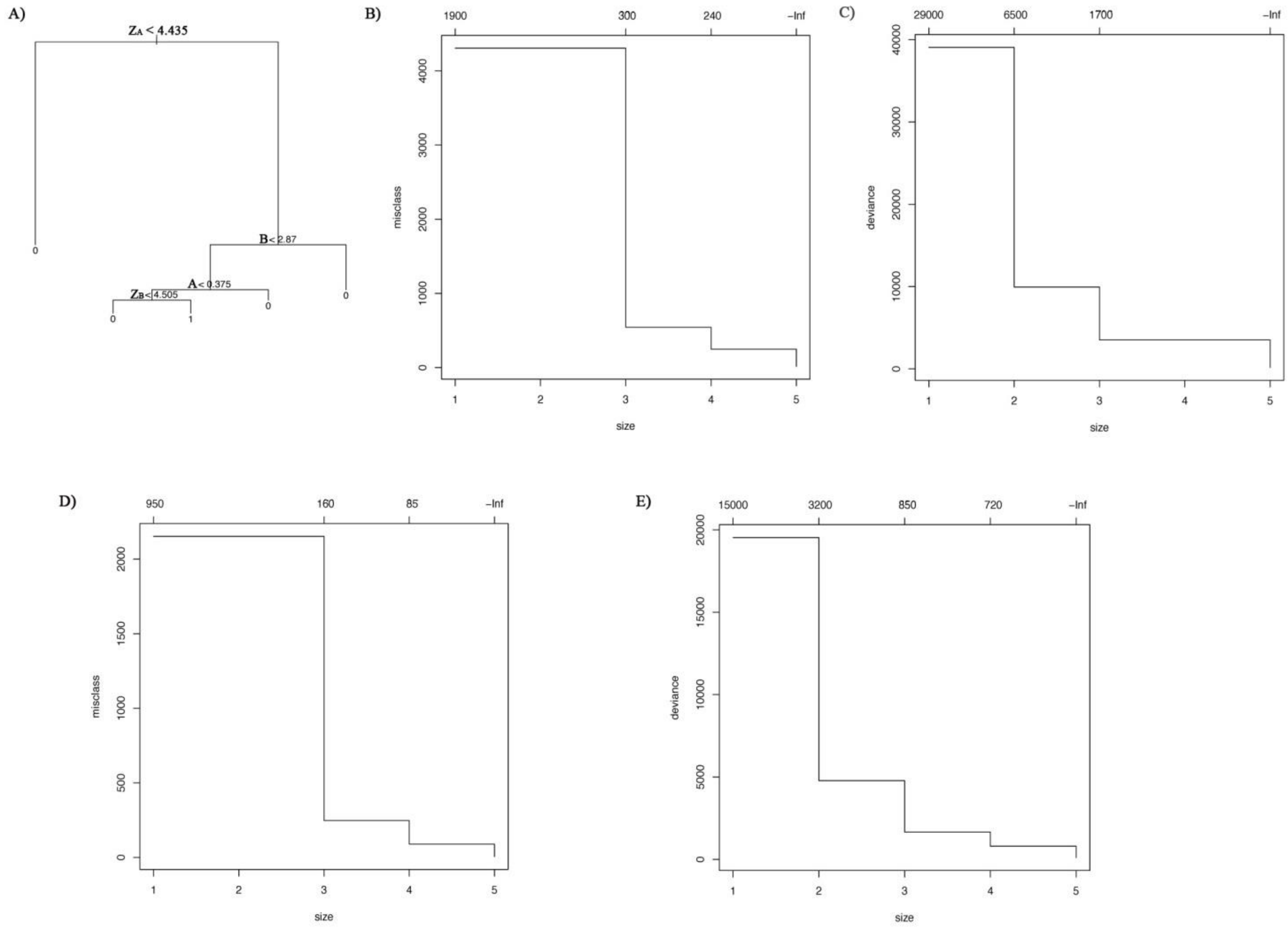

Suppl. Figure 1. Determination of parameter thresholds. A) Decision tree of the training sets. Both training sets produced the same decision tree. B) Misclassification plot from the decision tree obtained from all the training sets. C) Deviance plot from the decision tree obtained from all the training sets. D) Misclassification plot from the decision tree obtained from the subset of the training set. E) Deviance plot from the decision tree obtained from the subset of the training set.

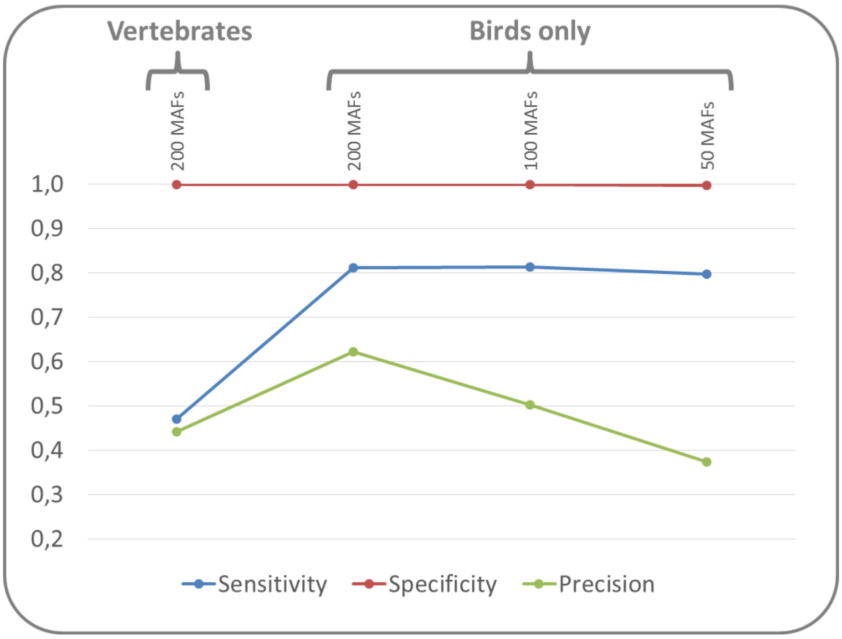

Suppl. Figure 2. Distribution of the sensitivity and specificity values for the different subsets. Increasing the divergence between the species reduces the sensitivity of the method, as it becomes more difficult to distinguish between true divergence and error. Using a larger number of multiple sequence alignment files (MAFs) containing the same species within the same divergence range, increases the precision of DivA, since the thresholds that determine the outlier windows are calculated using the parameter values from all the input alignments.

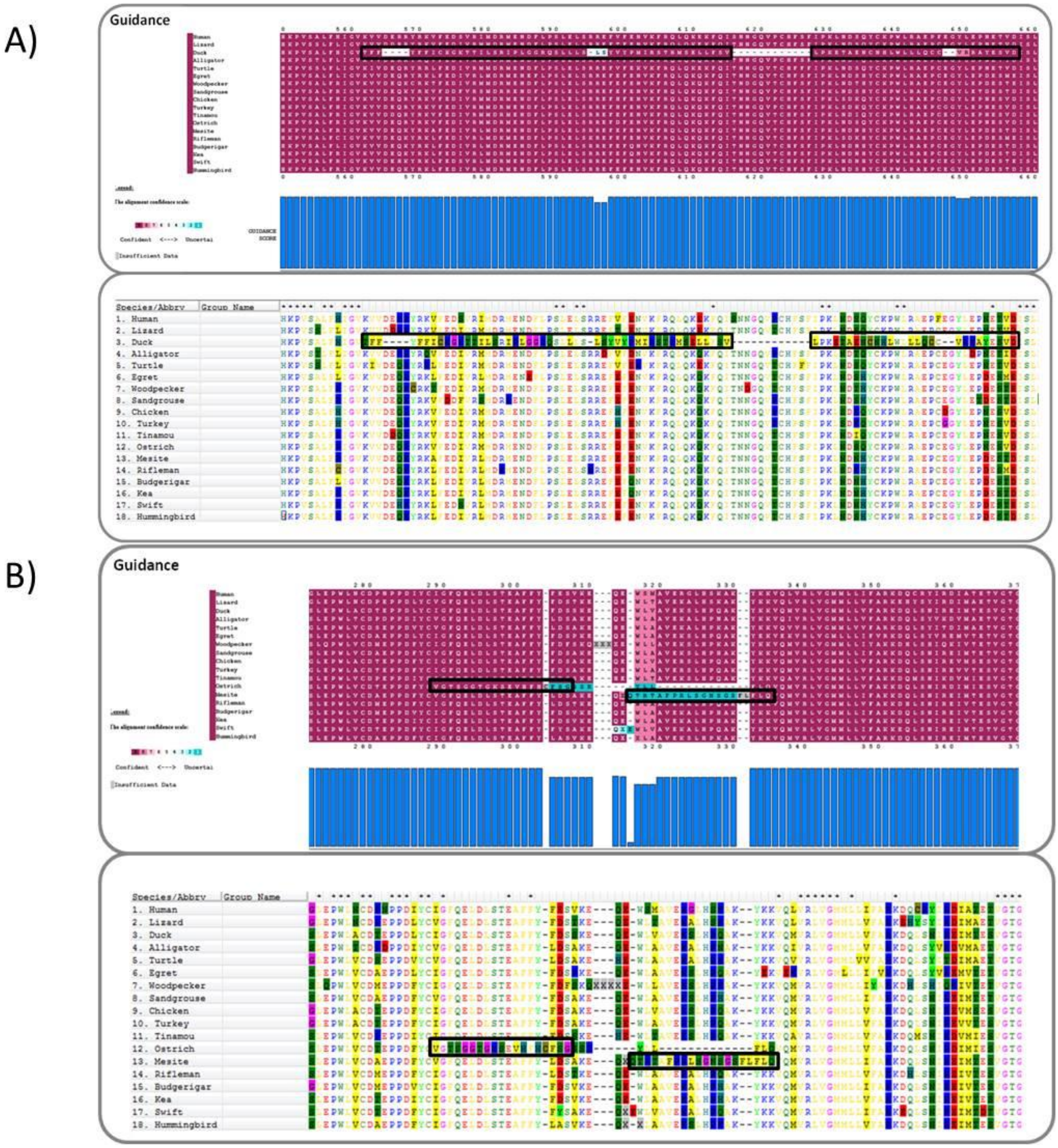

Suppl. Figure 3. Two regions of the Test MSA analyzed by DivA and Guidance. The alignment was done in Guidance [1] using Muscle [2] and DivA was run with the default parameters. The top parts of A and B display the output of the Guidance webserver [3]. Outlier windows determined by DivA are shown in black boxes. MEGA5 [4] was used to display the alignment view (the option to toggle off sites with a conservation score of more than 80% was used for an easier visualization of the outlier amino acids).

Suppl. Table 1. Amino acid conservation impact on model accuracy. Conservation scores per window were calculated as the total count of the most frequent amino acid divided by the total number of amino acids in each position of the alignment.

Alignment type		Conservation score	SD
highest TP	Complete alignment	0.9357	0.04
	Correctly classified windows	0.9365	0.02
highest FP	Complete alignment	0.91	0.104
	Incorrectly classified windows	0.851	0.164

References

1. Penn O, Privman E, Landan G, Graur D, Pupko T: **An alignment confidence score capturing robustness to guide tree uncertainty.** *Mol Biol Evol*. 2010;27(8):1759–67.

2. Edgar RC: **MUSCLE: multiple sequence alignment with high accuracy and high throughput.** *Nucleic Acids Res*. 2004;32(5):1792–7.

3. The GUIDANCE server. Available at: http://guidance.tau.ac.il/.

4. Tamura K, Peterson D, Peterson N, Stecher G, Nei M, Kumar S: **MEGA5: molecular evolutionary genetics analysis using maximum likelihood, evolutionary distance, and maximum parsimony methods.** *Mol Biol Evol*. 2011;28(10):2731–9.
